# Public perceptions and engagement with Traditional Chinese Medicine on Japanese social media (2010–2025): a text mining approach

**DOI:** 10.3389/fpubh.2026.1826864

**Published:** 2026-05-19

**Authors:** Shanshan Zhang, Xi Chen

**Affiliations:** 1 Graduate School, Xi‘an International Studies University, Xi'an, China; 2 School of Economics and Management, Xi‘an University, Xi'an, China; 3 School of Japanese Culture and Economics, Xi‘an International Studies University, Xi'an, China

**Keywords:** BERTopic, co-occurrence network, digital health, sentiment analysis, social media, Traditional Chinese Medicine (TCM)

## Abstract

Traditional Chinese Medicine (TCM) is a holistic medical system whose global visibility has increased markedly, yet large-scale studies on public perceptions and engagement remain limited. Using Japanese Twitter data from 2010 to 2025, this study employs text mining techniques including BERTopic modeling, sentiment analysis, and co-occurrence network analysis to examine public discourse on TCM. Building on the Cognition-Affect-Behavior (CAB) framework, this study employs multilevel relational analyzes to examine the interplay between topics, sentiment, and behavioral engagement. The results show that public discussions mainly focus on clinical efficacy and daily wellness, traditional knowledge and scientific innovation, business and cultural promotion of TCM. Despite the dominance of neutral and positive sentiment, negative sentiment shows an increasing trend. Concerns are related to scientific rigor, ecological ethics, and adverse experiences. Japanese users are most engaged with tweets about TCM wellness, ingredients, and therapies. These behaviors reflect patterns of active learning, cultural identity, and experiential exploration. This study provides empirical support for the CAB pathway in social media environments and elucidates both the diffusion patterns of TCM-related discourse and the mechanisms underlying public responses on Japanese social media. It offers theoretical and empirical implications for cross-cultural health communication and the analysis of TCM discourse in digital contexts.

## Introduction

As a cornerstone of Chinese civilization, Traditional Chinese Medicine (TCM) has evolved into a central pillar of China's global cultural outreach (1). Since acupuncture's inclusion on UNESCO's Intangible Cultural Heritage list in 2010 and Tu Youyou's 2015 Nobel Prize for artemisinin, TCM has gained growing global visibility and scientific interest. Yet its international rise has also intensified debate. Supporters view it as a “national medicine” symbol resisting foreign intrusion (2), whereas critics question its scientific validity and empirical verifiability (3, 4). This polarized communicative landscape shapes global perceptions of TCM and widens intercultural cognitive gaps. Therefore, effectively articulating TCM's values in forms intelligible and convincing to diverse global audiences has become an urgent yet unresolved challenge in its international dissemination.

Addressing this challenge requires moving beyond earlier Western-centric research paradigms and systematically examining public perceptions, sentiments, and behavioral responses toward TCM (5). Although research on the dissemination of TCM has been conducted across multiple fields and disciplines, existing studies still have limitations, primarily reflected in three research gaps.

First, existing research has mainly concentrated on discrete events or application scenarios and has not conducted user research from a holistic perspective. Prior investigations on user attitudes were limited to TCM in specific contexts, such as TCM tourism programs (6), virtual TCM exhibitions (7), acupuncture therapies (8, 9), TCM health initiative experiences (10), and the role of TCM in COVID-19 prevention and treatment (5, 11–13). Given that public attitudes toward TCM and its various scenarios differ, and that these attitudes play a key role in cross-cultural dissemination (14), it is necessary to comprehensively investigate public perceptions, attitudes, and focal interests regarding TCM. Second, most existing studies rely predominantly on user data obtained via surveys or in-depth interviews (15, 16), lacking verification and supplementation with objective data. Such subjective data are constrained by sample size, self-presentation biases, and other factors, making it difficult to fully reflect the public's authentic experiences in natural contexts (17). Third, existing research has largely focused on either cognitive or affective dimensions in isolation, leaving the systematic presentation of cognition, affect, and interactive behavior insufficiently explored (18, 19). Based on the Media Effects Model, public cognition, emotional preferences, and behavioral intentions serve as key indicators for assessing the effectiveness of cultural and informational dissemination (20). Therefore, to optimize cross-cultural TCM dissemination, larger-scale and more objective datasets are needed to systematically investigate cognition, sentiment, and behavior in an integrated manner.

As China's close neighbor, Japan occupies a distinctive position within the East Asian cultural sphere, embodying both modern and traditionally Eastern characteristics in its medical technologies and cultural perceptions (21). Within this dual identity, Japanese public attitudes and behaviors toward TCM can provide a unique perspective for understanding the cross-cultural dissemination of TCM in East Asia and beyond. In recent years, social media platforms—particularly Twitter —have emerged as key arenas for the Japanese public to articulate opinions, disseminate health knowledge, and engage in cross-cultural discourse (22, 23). Within the user-generated content (UGC) paradigm, users act as both content consumers and producers, breaking the traditional one-way media communication model and significantly enhancing the impact and effectiveness of cultural dissemination (24).

Building upon these considerations, this study systematically analyzes public discourse on TCM on Japanese Twitter from 2010 to 2025 from the perspective of UGC text mining. The specific research questions are as follows:

RQ1. What are the dominant topics, concepts, and contents on Twitter in Japan regarding TCM?

RQ2. What are the sentimental inclinations of online Japanese users toward TCM?

RQ3. How do discussions of TCM on Twitter shape Japanese users' social media behaviors?

**Figure 1:**
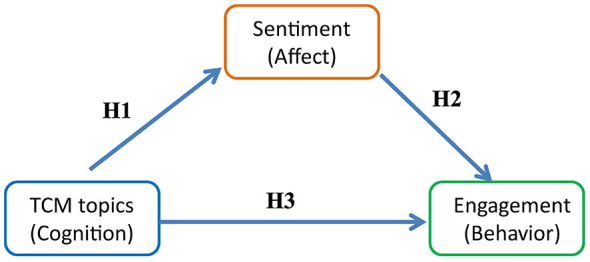
CAB-based conceptual framework for TCM-related discourse on Japanese social media.

In light of the above research questions, this study adopts the Cognition–Affect–Behavior (CAB) framework to investigate the mechanisms underlying the dissemination of TCM-related discourse on Japanese social media (25). As illustrated in Figure 1, its operationalization within social media analytics enables topic clusters to proxy the public's cognitive representations of TCM, sentiment signals to capture affective orientations, and engagement metrics—such as likes, replies, and retweets—to index observable behavioral responses. On this basis, cognition shapes behavior both directly and indirectly via affect, forming a “topic–sentiment–engagement” pathway.

Guided by this theoretical framework and building on the research questions outlined above, this study formulates the following hypotheses:

**H1:** Sentiment differs significantly across TCM-related topics.

**H2:** Affective orientations significantly influence user engagement and mediate the relationship between topics and user engagement.

**H3:** TCM-related topics exhibit significant variation in both the intensity and structure of user engagement (i.e., the relative proportions of likes, replies, and retweets).

The main contributions of this study are as follows. First, it leverages advanced large language models to capture the cognitive dynamics of the Japanese public toward TCM-related topics based on user-generated content, addressing a gap in the existing literature. Second, it traces temporal shifts in public sentiment, providing a novel perspective on cross-cultural health communication. Third, drawing on the CAB framework, it elucidates the interplay among cognition, affect, and behavior in social media contexts, thereby revealing the diffusion patterns of TCM-related discourse and the mechanisms underlying public engagement.

## Literature review

### Media studies on TCM

Media serves as a vital conduit for disseminating and popularizing TCM, playing a pivotal role in shaping its modern interpretation and global reach. Existing research has predominantly examined how mainstream media shape the portrayal of TCM and deploy discourse strategies. For instance, Zhang et al. examined global media coverage of TCM between 2018 and 2024, revealing pronounced shifts in emotional agenda-setting before and after the pandemic (19). Their findings indicate that cultural distance exerts a nuanced effect, where greater cultural divergence may amplify both the appeal and impact of emotional agendas. Chen et al. used the Nobel Prize awarded to Chinese pharmacologist Tu Youyou as a case, analyzing 418 posts on social media published by official media and medical experts about TCM (18). The results showed that, despite the diversified space for opinion expression on Weibo, mutual understanding and integration among different groups did not improve, leading instead to fragmentation of the public sphere. Zhu analyzed TCM discourse in Chinese mainstream television, showing that the media, by reinforcing expert authority and emphasizing traditional expectations and moral norms, subtly influence individual behavior while reinforcing mainstream culture and political ideology, leaving little room for challenging dominant norms (26). Similarly, Zhou focused on social media accounts of official TCM institutions, using critical discourse analysis and textual analysis to conduct an interdisciplinary study on symbolic construction practices related to COVID-19 TCM treatment (27).

### Public attitudes and emotions toward TCM

At the public level, research has focused on how individuals perceive and emotionally respond to Traditional Chinese Medicine (TCM), addressing both cognitive and affective dimensions. In the cognitive dimension, Duan et al. found that the public's overall acceptance of TCM services was high, yet actual utilization varied: 97.33% of respondents had received TCM services, but usage rates for acupuncture, massage, cupping, and other treatments were generally low, indicating a discrepancy between cognition and behavior (28). Wang et al. noted that economic and political factors exert a significant influence on Chinese public perceptions of traditional TCM (13), while Liu and Zhang further highlighted that multiple factors—including cross-disciplinary discussions, expressions of national identity, the use of uncivil language, and discussion depth—also shape public cognition (5).

In the affective dimension, several studies have reported pronounced emotional polarization in online discussions of TCM topics (5, 11, 12). Gao et al. analyzed TCM-related posts on Weibo during the COVID-19 pandemic, revealing that 12% of posts conveyed positive attitudes toward TCM, 10% conveyed negative attitudes, and 78% were neutral (29). Positive emotions have been closely linked to factors such as cultural pride, trust in scientists and healthcare professionals, and perceived value (15, 18, 28), whereas negative emotions primarily arise from insufficient scientific evidence, limited treatment efficacy, and low levels of policy trust (3, 16).

Existing research has examined TCM across contexts ranging from media discourse to public expression. However, firstly, most studies focus on specific events or scenarios and rely primarily on small-scale surveys, resulting in limitations in sample size, subjectivity, and scope of investigation. Second, existing studies tend to examine cognitive or affective dimensions in isolation, with limited integration of cognition, affect, and interactive behavior. Positive emotions among the public can promote interactive behaviors, including information uptake, knowledge sharing, and cultural dissemination, whereas negative emotions may trigger cognitive resistance, information avoidance, and even herd-like patterns of adverse dissemination (30). Therefore, this study aims to systematically analyze TCM-related posts on Japanese Twitter using big data processing tools and computational techniques (topic modeling, sentiment analysis, co-occurrence network analysis), thereby filling existing research gaps. By adopting this multidimensional methodology, the study seeks to uncover how Japanese public cognition, affective responses, and behavioral intentions collectively shape perceptions of TCM, thereby offering novel empirical insights into the mechanisms of its cross-cultural dissemination.

## Data and methodology

### Research design

**Figure 2:**
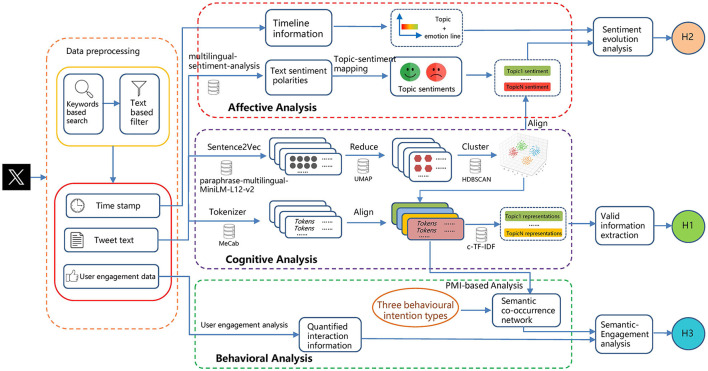
Research framework.

This study examines TCM-related posts on Japanese social media, systematically investigating the interplay among public cognition, affective responses, and behavioral intentions toward TCM. The overall research design framework of this study is shown in Figure 2.

### Data collection and preprocessing

With the evolution of Japan's social media ecosystem, platform diversity has expanded markedly, driven by the proliferation of platforms such as Instagram, Facebook, and TikTok. However, these platforms vary substantially in content formats and user bases. Instagram, despite its 2010 launch, saw limited early adoption in Japan and, as a visually oriented platform, offers relatively sparse textual data, making it ill-suited for constructing a longitudinal, text-rich corpus (2010–2025). TikTok, introduced in 2017, is inherently video-centric, with fragmented textual signals that constrain its suitability for systematic discourse analysis. Facebook, while supporting multimodal communication, relies on egocentric networks that limit information diffusion within semi-closed structures, thereby restricting its capacity to capture large-scale public discourse. Other platforms, including Reddit, and YouTube, present similar limitations, rendering them less suitable for large-scale longitudinal corpus construction.

In contrast, Twitter (now X), as one of the most widely used social media platforms in Japan, plays a central role in public discourse and information diffusion. Its short-form textual architecture enables a high degree of openness and immediacy, making it particularly well suited for large-scale text mining and longitudinal analyzes of topic evolution and sentiment dynamics. Twitter had approximately 75.8 million users in the country by 2025, accounting for about 60% of the national population (31). Accordingly, compared to other platforms, Twitter provides a more representative data source, enabling a comprehensive and less biased account of the Japanese public's salient concerns and affective orientations toward TCM-related topics. Building on this, the study collected longitudinal Twitter data spanning 2010–2025 on public discussions of TCM in Japan and performed systematic data preprocessing. The specific steps are as follows:

(1) **Data collection.** We used the Twitter API to retrieve relevant Japanese-language tweets and construct the corpus. To maximize retrieval coverage of TCM-related content, we selected keywords based on relevant literature and preliminary observations of Twitter posts. The language was restricted to Japanese, and the search terms included “中医” (Traditional Chinese Medicine), “漢方” (Kampo), “漢方薬” (Kampo medicine) , “東洋医学” (East Asian medicine), “伝統医学” (traditional medicine). The dataset covers the period from January 2010 to December 2025. For each tweet, we recorded the user ID, posting time, username, display name, tweet content, number of likes, retweets, and replies, resulting in a total of 58,725 tweets.

It should be noted that the selection of keywords in this study was intended to comprehensively capture public discussions of TCM in Japan. However, “Kampo” (漢方) has developed into a distinctly localized medical system, meaning that the resulting dataset may extend beyond TCM in the strict sense to encompass broader traditional medical systems.

(2) **Data cleaning and preprocessing.** After collecting the raw tweets, preprocessing was conducted in several steps. First, HTML tags, URLs, emojis, and extra whitespace were removed, and full-width and half-width characters were normalized. Second, the tweet text and timestamps were retained, and key information was extracted. Subsequently, the MeCab tokenizer (with IPA, UniDic, and Juman dictionaries) was used for Japanese tokenization. Finally, a dataset containing 53,983 valid entries was constructed.

### Methodology

#### Topic Mining Based on BERTopic

This study employed the BERTopic large language model for topic mining. BERTopic is a BERT-based topic modeling approach that integrates UMAP for dimensionality reduction and HDBSCAN for density-based clustering, while leveraging c-TF-IDF strategy to generate semantically coherent and highly representative topic labels (32). The approach excels at capturing high-dimensional embeddings and complex, nonlinear semantic relationships, making it especially well-suited for unstructured, short-text, multilingual social media data (33, 34). The workflow for applying the BERTopic model is as follows:

(1) **Text embedding generation.** We employed the paraphrase-multilingual-MiniLM-L12-v2 model (35) to generate dense embedding vectors from the pre-processed tweets. This model is a sentence-transformers model that generates embedding vectors for the entire text from a sentence semantic perspective, ensuring semantic integrity.
(2) **Dimensionality reduction.** To improve visualization and clustering efficiency, the UMAP algorithm was used to perform nonlinear dimensionality reduction on the generated high-dimensional semantic vectors (36). The n_neighbors parameter was set to 20 to balance local and global feature preservation.
(3) **Text clustering.** For the dimensionally reduced vectors, we used the HDBSCAN model to perform density-based clustering. This model can automatically determine the optimal number of clusters and identify some discrete texts as “noise” without forcibly classifying them, enhancing the scientific rigor of topic delineation (37). Finally, the model identified 8 main topic clusters, each representing a set of tweets with highly similar content.
(4) **Topic representation and optimization.** To enhance the interpretability of each topic, the study introduced the c-TF-IDF algorithm to model and reinforce topics (32). Specifically, all tweets within each topic cluster were concatenated into a single document, from which word frequencies were computed and contrasted against the frequency distribution across the entire corpus to identify the most representative terms for each topic. This method can effectively highlight the unique semantic features of each topic and avoid interference from noise words.

To improve both the diversity and semantic coherence of extracted keywords, we incorporated the Maximum Marginal Relevance (MMR) mechanism, which balances the trade-off between term relevance and redundancy. Specifically, MMR employs a tunable weighting parameter to retain representative keywords while suppressing semantically redundant or overlapping terms, thereby enhancing information coverage and improving the distinctiveness and interpretability of topic representations.

To evaluate topic clustering performance, we randomly sampled 3% of the corpus and conducted manual annotation with three Japanese-language experts. The results show strong agreement between BERTopic-derived classifications and human annotations, achieving 92.23% accuracy and an F1 score of 0.91, highlighting the model's effectiveness in capturing and differentiating thematic structures.

#### Sentiment analysis

To investigate Japanese public attitudes toward TCM and their evolution, we employed the multilingual-sentiment-analysis model to assess the sentiment of each tweet (38). The model is based on the mBERT architecture and pre-trained on large-scale multilingual corpora, capable of capturing fine-grained sentiment features with high accuracy. Each tweet was classified into one of five sentiment categories (very negative, negative, neutral, positive, or very positive), accompanied by a confidence score. To validate sentiment classification performance, we randomly sampled 3% of the dataset and conducted independent sentiment annotation with three Japanese-language experts. The model achieves 90.35% accuracy and an F1 score of 0.89 against human annotations, demonstrating strong agreement. These results confirm the model's robustness in processing Japanese tweets and its suitability for longitudinal sentiment analysis of public discourse. By performing sentiment classification on each tweet and conducting further statistical analysis, we quantified public opinions on TCM and examined the evolution and fluctuation of public emotions within each topic. By integrating the results of sentiment analysis, this study aims to provide a comprehensive interpretation of Japanese public attitudes toward TCM on Twitter, thereby elucidating the dissemination dynamics and social media impact of TCM.

#### Co-occurrence network analysis

To explore the potential behavioral intentions of the Japanese public toward TCM, this study employs co-occurrence network analysis on tweets and their associated interaction content. First, tweets with at least ten replies were retained to ensure adequate interactional representativeness. The numbers of likes, retweets, and replies were then used as indicators of user engagement (39). Drawing on social cognitive theory and cross-cultural communication theory, potential behavioral intentions were categorized into three dimensions: active learning, cultural identification, and experiential exploration , capturing tendencies for knowledge acquisition, value resonance, and immersive experiential engagement (40, 41). Methodologically, co-occurrence network visualization was employed to illustrate how key contents in tweets and replies co-occur across the three dimensions of behavioral intention. Specifically, keywords associated with the three behavioral dimensions were extracted as network nodes, and edges were established according to the co-occurrence relationships among these nodes (42). Finally, analysis of the network's clustering structure and node distribution reveals the cognitive focal points and behavioral intention patterns underlying public engagement with TCM culture.

We adopted a Pointwise Mutual Information (PMI)-based method to calculate the co-occurrence relationships among keywords and constructed a semantic co-occurrence network.

Specifically, apart from the three predefined behavioral intention terms, the size of the other nodes in the graph is represented by the product of normalized word frequency intensity and average interaction intensity, calculated using the following formula:


$$\begin{array}{l} NodeSize\left(w\right)=Freq\left(w\right)\times AvgEng\left(w\right) \end{array}$$ (1)

*Freq*(*w*) represents the total number of occurrences of word *w* in the tweets (i.e., the total word frequency), while *AvgEng*(*w*) denotes the average engagement intensity of word *w*. The calculation of *AvgEng*(*w*) is as follows:


$$\begin{array}{l} AvgEng\left(w\right)=\frac{\sum_{t\in T_{w}}\left(\alpha \cdot Like_{t}+\beta \cdot Retweet_{t}+\gamma \cdot Reply_{t}\right)}{DF\left(w\right)} \end{array}$$ (2)

In Equation 2, the numerator represents the total engagement intensity associated with word *w*, while the denominator *DF*(*w*) denotes the number of tweets containing the word.

*T*_*w*_ represents the set of tweets containing word *w*. *Like*_*t*_, *Retweet*_*t*_, and *Reply*_*t*_ denote the numbers of likes, retweets, and replies of tweet *t*, respectively. Based on the intensity characteristics of interaction behaviors, this study sets α = 0.25, β = 0.35, and γ = 0.40.

After normalizing the results of the above calculations, the size of each node word is obtained.

The edges between nodes are computed as follows: First, calculate the probability of each word and the joint probability of two words, as follows:


$$\begin{array}{l} P\left(w\right)=\frac{f\left(w\right)}{N},P\left(v\right)=\frac{f\left(v\right)}{N},P\left(w,v\right)=\frac{f\left(w,v\right)}{N} \end{array}$$ (3)

*N* denotes the total number of tweets; *f*(*w*) represents the number of tweets containing word *w*; *P*(*w*) is the probability of word *w* occurring; *f*(*w, v*) represents the number of tweets containing both words *w* and *v*; and *P*(*w, v*) is the probability of co-occurrence of *w* and *v*.

The PMI between words *w* and *v* can therefore be computed as follows:


$$\begin{array}{l} PMI\left(w,v\right)=\log\frac{P\left(w,v\right)}{P\left(w\right)P\left(v\right)} \end{array}$$ (4)

In this study, the edges are defined as follows:


$$Edge_{w,v}=\{\begin{array}{c} 1,\;if\;PMI(w,v)>0\\ 0,\;otherwise \end{array}$$ (5)

Only when the *PMI*(*w, v*) coefficient is greater than 0 is the edge between word *w* and word *v* retained.

## Results

### Public attention topics on TCM

**Figure 3:**
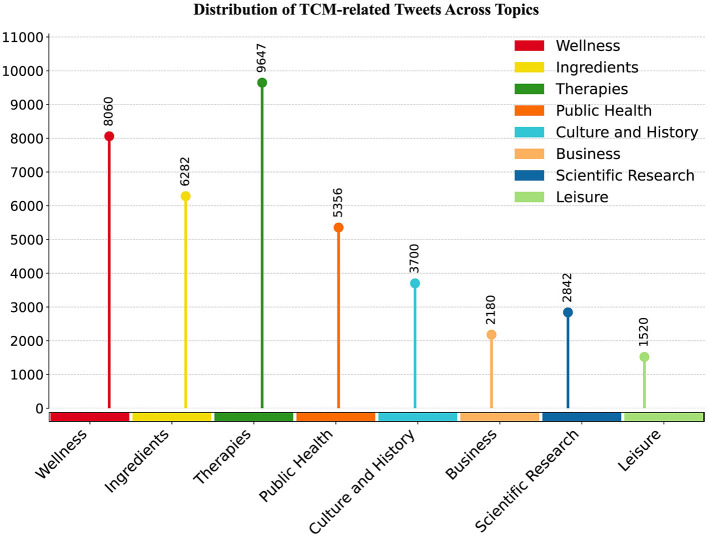
Distribution of TCM-related tweets across topics.

This study applied BERTopic modeling to the cleaned and collected tweets, extracting and analyzing topics based on visualization results. Ultimately, eight main research topics were automatically identified from 53,983 valid tweets. The topic extraction results are shown in Table 1. Figure 3 illustrates the distribution of tweet volumes across different TCM-related topics.

**Table 1:** BERTopic topic extraction results.

Topic ID	Category	Representative keywords
Topic 0	Therapies	0_中医_漢方_施術_薬方
Topic 1	Wellness	1_健康_茶_ハーブ_薬膳
Topic 2	Ingredients	2_生薬_薬剤_成分_作用
Topic 3	Public Health	3_コロナ_予防_感染_肺炎
Topic 4	Culture and History	4_歴史_伝統_医書_陰陽
Topic 5	Scientific Research	5_科学_学者_大学_試験
Topic 6	Business	6_薬局_供給_会社_販売
Topic7	Leisure	7_ドラマ_映画_シリーズ_時代劇

**Figure 4:**
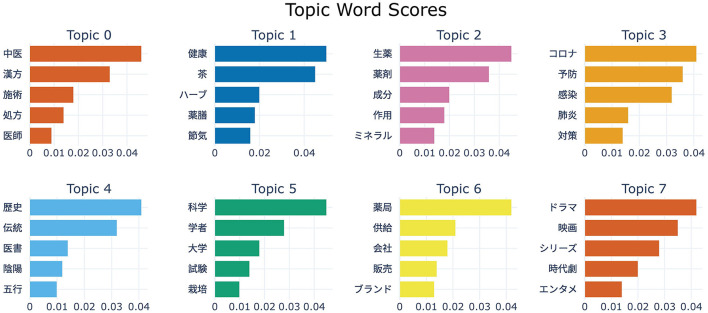
Score map of topic feature words.

To provide a clearer visualization of term significance across topics, we computed the c-TF-IDF scores for each term, as depicted in Figure 4. Here, the length of each bar corresponds to the term's c-TF-IDF score, offering an immediate visual representation of the relative importance of terms within their respective topics.

Based on the results of BERTopic modeling, we aggregated the eight topics into three overarching dimensions: (1) clinical efficacy and daily wellness (Topics 0–2), (2) traditional knowledge and scientific innovation (Topics 3–5), and (3) business and cultural promotion (Topics 6–7), as summarized below.

The most prominent dimension is clinical efficacy and daily wellness (Topics 0–2), regarding TCM's practical effectiveness, health principles, and safety considerations. First, the global expansion of TCM is driven by the pressing need for alternative therapies among individuals with chronic conditions. By browsing through the user posts, it can be observed that many Japanese express both concern and anticipation regarding TCM's actual efficacy in managing chronic illnesses. For example, one user mentioned: “Someone said that sleeping with mouth breathing causes a sore throat, so a friend gave me some Chinese medicine? I heard it's very good.” Another user shared: “Paeoniae Radix, is a Chinese medicinal herb effective for headaches, back pain, and gynecological diseases.” Public discussions mainly focus on TCM's role in managing chronic diseases, women's health care, and the potential benefits of traditional therapies such as acupuncture, massage, and qigong in alleviating pain and promoting recovery.

Second, the public shows strong interest in TCM's role in daily health maintenance. Discussions around dietary therapy, seasonal wellness, and beauty health are particularly frequent, such as many tweets mentioning “TCM fugu hotpot” or “eating pomegranate in autumn.” Such discussions are closely tied to daily life, demonstrating recognition of maintaining physical and mental health through diet. They also reflect the penetration of TCM's preventive treatment concept in Japanese society.

Third, safety and ethical concerns are increasingly prominent. Keywords such as “ingredients,” “effects” frequently appear in tweets, reflecting public concerns about medicinal material sourcing and animal protection. For example, many users noted: “many animals and plants have become endangered species due to the use of Chinese medicine.” Additionally, users expressed concerns regarding the reliability of TCM efficacy and quality standards, exemplified by comments such as “The efficacy of Chinese medicine varies from person to person” and “What are the safety standards for imported Chinese medicine?” These discussions indicate that the Japanese public exercises careful scrutiny over ethical practices, standardization, and safety issues in the global dissemination of TCM.

The dimension of traditional knowledge and scientific innovation (Topics 3–5) involves public discussions on pandemic control, the history of traditional medicine, and modern scientific innovation, showing the discourse transformation of TCM in Japanese society from a “traditional heritage” to a “scientific resource.” First, Topic 3 (Public health) reflects the practical concerns of TCM in the context of global health crises. Users frequently mentioned terms such as “COVID-19”, “prevention”, and “measures” to express their interest in the preventive and therapeutic potential of TCM during the pandemic. For instance, one user remarked, “Reports indicate that 97% of COVID-19 patients in China achieved positive outcomes with TCM. According to the Japan Society of Chinese Medicine, specific herbal formulas can be selected according to patient symptoms.” Another user observed, “Many TCM products used in Japan are imported from China. Those believed effective against COVID-19 have experienced skyrocketing demand in China, which could potentially lead to supply shortages in Japan.” These comments suggest that Japanese engagement with TCM during health crises is characterized by a curious and exploratory stance.

Topics 4 and 5 indicate that the public not only recognizes the cultural heritage value of TCM, but also pays attention to its modernization and scientific development. Keywords such as “history”, “tradition”, “science”, and “experiments” frequently appear in the tweet corpus. These keywords reflect the public's perception of integrating traditional TCM knowledge with evidence-based scientific systems, as well as their expectations for the scientific and standardized development of TCM.

The dimension of business and cultural promotion (Topics 6–7) illustrates how TCM extends into the realm of cultural consumption. On the one hand, the public shows strong interest in enterprises engaged in TCM research and production. Tweets indicate that although Chinese companies such as “Beijing Tongrentang” actively promote their products on Japanese social media platforms, the Japanese public places significantly higher trust in local brands such as “Tsumura” than in “Made in China” products. This suggests that brand image and quality standards play a crucial role in the cross-cultural dissemination of traditional Chinese medicine. Although TCM is widely recognized as originating in China, underlying distrust toward product quality in other industries may spill over subconsciously, leading some consumers to prefer domestic brands when choosing TCM-related products. On the other hand, TCM elements frequently appear in films, TV series, and advertisements, serving as prominent symbols that embody Chinese cultural imagination. Motifs such as “imperial physician” and “pulse diagnosis” in historical dramas or suspense series transform TCM from purely medical knowledge into a cultural narrative resource, facilitating public understanding of Chinese culture through media experiences.

The observed distributional patterns of topic structures extend beyond simple measures of discussion intensity, revealing a pragmatic orientation in the circulation of TCM within Japanese society. First, the prominence of “clinical efficacy” points to a compensatory turn toward alternative medicine amid the rising burden of chronic disease, alongside the diffusion of the TCM principle of preventive care (“treating illness before its onset”). Second, the growing emphasis on evidence-based validation reflects the integration of traditional medicine into modern regimes of scientific evaluation and legitimacy. Finally, despite its strong cultural-symbolic resonance, trust in TCM-related brands exhibits a clear localization bias toward domestic Japanese products, indicating that perceived product quality remains the primary criterion guiding user choice in cross-cultural contexts. Taken together, these findings suggest that the global diffusion of TCM is shaped by the interplay of pragmatic motivations, scientific rationality, and product quality evaluations.

### Sentiments analysis and focus

#### Sentiment tendency

This study employed a pre-trained multilingual sentiment analysis model to perform fine-grained sentiment classification of the collected TCM-related tweets, categorizing them into five classes: very negative, negative, neutral, positive, and very positive. Table 2 shows the overall sentiment distribution of TCM-related posts from 2010 to 2025. As shown in Table 2, neutral and positive sentiment postings dominate the distribution, together comprising 74.21%. Notably, positive sentiment (positive plus very positive) represents the largest proportion at 43.43%. A closer inspection reveals a clear polarity asymmetry: very positive posts (29.02%) markedly exceed positive ones (14.41%), while very negative posts (16.05%) similarly surpass negative ones (9.74%). This pattern aligns with prior findings (18), suggesting that social media users are more inclined to express strong affective stances—either positive or negative—rather than moderate evaluations when discussing TCM.

**Table 2:** The overall sentiment distribution of TCM-related posts.

Very negative	Negative	Neutral	Positive	Very positive
16.05%	9.74%	30.78%	14.41%	29.02%

This polarized distribution of sentiment is consistent with self-presentation theory, whereby users strategically adopt highly positive or highly negative stances on social media to signal identity and reinforce group belonging (43). As a culturally embedded medical system, TCM readily elicits affective involvement, shifting discourse from ostensibly objective medical evaluation toward more subjective and emotionally charged communication. This polarization further reflects the amplification of extreme emotions on social media, where very positive and very negative content diffuses more widely than moderate attitudes and is more likely to elicit user engagement.

**Figure 5:**
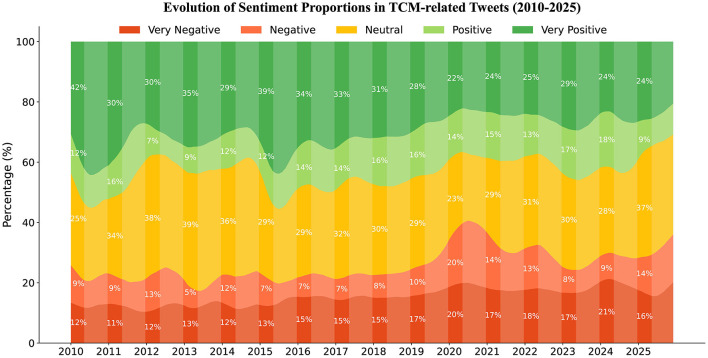
Evolution of sentiment proportions in TCM-related tweets (2010–2025).

Figure 5 shows the annual evolution of the five sentiment categories from 2010 to 2025. It can be observed that although neutral and positive sentiments dominate, their proportion shows a decreasing trend over the years. In contrast, negative and very negative sentiments, though relatively low overall, show a gradually increasing trend, rising from 21% in 2010 to 30% in 2025. Notably, following the outbreak of the COVID-19 pandemic in Japan in 2020, the proportion of negative sentiments regarding TCM increased significantly, and public attitudes toward TCM changed more dramatically. Manual review of relevant posts during the pandemic shows that, although many Japanese regarded TCM as a potential option for combating the pandemic, some posts expressed skepticism and negative evaluations, which may relate to cross-cultural cognitive differences and ideological biases.

#### Topic–sentiment association analysis

**Figure 6:**
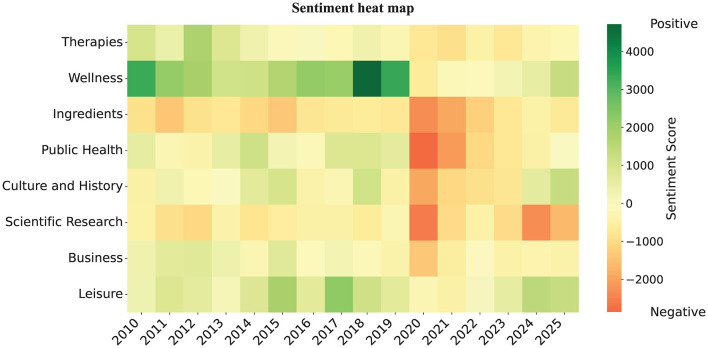
Topic–sentiment evolution heat map across TCM-related topics on Japanese social media. It presents a sentiment heat map organized by year and topic. Color intensity reflects the sentiment score, calculated annually by summing the sentiment values of all tweets within each topic (very negative = −2 negative = −1, neutral = 0, positive = +1, very positive = +2). Darker colors indicate stronger sentiment polarity, whether positive or negative. The horizontal axis represents the year, while the vertical axis lists the 8 topics.

To examine the association between TCM-related topics and sentiment, this study analyzes sentiment distribution across topics and traces its temporal evolution, as visualized in a topic–sentiment evolution heatmap (Figure 6).

As shown in Figure 6, sentiment distributions vary substantially across topics. The “Wellness” and “Leisure” topics are predominantly associated with positive sentiment, indicating that TCM is often framed as a source of wellbeing and emotional comfort, thereby fostering user identification and positive evaluations. In contrast, the “Ingredients” and “Scientific Research” topics display a higher concentration of negative sentiment, reflecting concerns over ingredient controversies, reliance on herbal resources, and elevated risk perceptions.

The figure reveals substantial variation in public sentiment across TCM-related topics. While lifestyle-oriented domains (Therapies, Wellness, Leisure) are associated with positive evaluations, discourse in public and professional domains (Scientific Research, Ingredients, Public Health) is more often marked by skepticism and distrust. This divergence suggests that the diffusion of TCM in Japan is constrained less by limited cultural appeal than by perceived shortcomings in the transparency of scientific standards and the credibility of ethical and safety assurances.

Furthermore, sentiment evolution reveals substantial temporal variation across topics between 2010 and 2025. From 2010 to 2019, most topics were characterized by positive or neutral sentiment, indicating relatively stable and moderate affective patterns. “Wellness” and “Leisure” consistently exhibited high positive scores. During this period, TCM was frequently framed as a form of cultural heritage, with discourse centered on health and leisure, and public understanding remaining diffuse yet broadly positive. A clear inflection point emerges after 2020, as many topics shift toward negative sentiment or display intensified negativity, signaling an overall decline. This pattern suggests that, in the context of the COVID-19 public health crisis, TCM discourse moved from private wellness domains to public health arenas, prompting heightened scrutiny of its safety and scientific validity. Notably, sentiment in “Wellness” and “Leisure” rebounds after 2023, reflecting the resilience of public attitudes toward everyday health maintenance and leisure practices.

Taken together, the foregoing analyzes demonstrate substantial variation across TCM-related topics in both sentiment distributions and temporal dynamics, indicating that topical content plays a key role in shaping affective expression. These results provide strong empirical support for H1, which posits that sentiment distributions vary significantly across topics.

#### Sentiment focus analysis

**Figure 7:**
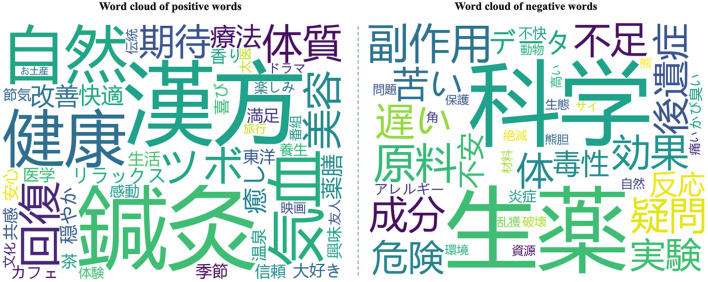
Word cloud of positive as well as negative words of the extracted tweets.

To comprehend the specific reasons behind these sentiments, this study utilized the Python TextBlob package to categorize words into positive and negative sentiment classes and generated corresponding word clouds. Figure 7 depicts the classification of words according to their sentiments. The word clouds show that *Kampo, Acupuncture*, *Health *and *Natural *are the most commonly occurring positive words, whereas *Science*, *Raw medicinal* *materials*, *Ingredients*, and *side effects *are the most commonly occurring negative words.

Users expressing positive attitudes toward TCM often attribute their sentiments to the pleasurable experiences derived from daily activities, such as acupuncture, seasonal wellness practices, and medicinal cuisine. Users frequently express joy by sharing personal experiences of TCM practices and health improvements. This frequently mentioned reason highlights a widespread perception among the Japanese public that TCM provides functional benefits for daily health management and holistic wellness. This finding is consistent with prior studies, suggesting that active participation in diverse TCM practices substantially enhances the perceived authenticity and enjoyment of TCM (7, 15). For instance, one user commented: “I received lotus seeds as a travel gift from a friend in China, so I tried making sweet boiled lotus seeds… They taste like chestnuts—very delicious! I understand that lotus seeds are also used as a medicinal ingredient in TCM, believed to nourish the heart, calm the mind, and alleviate fatigue. It seems they are also available for purchase online.”

Notably, in addition to the functional benefits of wellness practices, positive attitudes among the Japanese public are also shaped by the entertainment and cultural appeal of TCM. The popularity of Chinese TV dramas featuring TCM elements in Japan enables viewers to encounter and appreciate the symbolic significance of TCM, thereby eliciting emotional resonance. For example, a user commented: “It's those large pills often seen in Chinese TV dramas. I have been consuming them like a nutritional drink since childhood, so it doesn't seem strange at all… only now the price has increased and they are very expensive.” Another user expressed surprise at experiencing TCM diagnosis in person after seeing it on TV: “TCM doctors can diagnose diseases through pulse reading. Although it appeared many times in dramas, I thought ‘No way~?' but when I visited a TCM internal medicine clinic in Taipei, I realized it really works! The doctor was amazing!” These examples indicate that media portrayals of TCM can spark public interest, which, when reinforced by firsthand experience, translates into trust and positive attitudes toward TCM. Moreover, expectations regarding the efficacy of TCM are also an important source of positive attitudes. For example, a user stated: “I started taking Ling Gui Zhu Gan Tang, stopped all previous medications, and only took Zhen Wu Tang. I am very interested in how Chinese TCM approaches and treats Ménière's disease.” It can be seen that, for Japanese users, TCM—viewed as a symbol of “Eastern wisdom”—may carry heightened emotional significance.

The word cloud of negative sentiment indicates that various types of negative sentiments are elicited by distinct factors. As shown in Figure 7, users' negative sentiments are predominantly rooted in skepticism about the efficacy of TCM. Given TCM's distinctive theoretical framework and its strong reliance on clinical experience, its diagnostic precision and therapeutic consistency are frequently questioned (13), leading some users to regard it as “unscientific.”

Moreover, concerns regarding environmental degradation and animal welfare issues associated with TCM constitute significant sources of negative sentiment among the public. One user remarked: “Although licorice grows wild in certain regions of China, rising demand has raised concerns that overharvesting could cause soil degradation and depletion of natural resources.” Another user mentioned: “In recent years, poaching of rhinos in South Africa has dramatically increased to supply rhino horns. This surge has been attributed to rising demand for rhino horn as a prized TCM ingredient in China and Southeast Asia.” Such discussions indicate that certain segments of the Japanese public are apprehensive about the sustainability of Chinese medicine and the ethical implications for wildlife conservation.

In contrast to positive sentiments primarily arising from enjoyable experiences, negative sentiments may be elicited by adverse personal encounters. For instance, some users highlighted the issue of taste: “Traditional Chinese Medicine is very bitter; I cannot drink it.” Another user reported adverse reactions following acupuncture at the Chinese Kampo Tsuru Clinic in Nishi-Shinjuku, noting: “I went to an acupuncture clinic for an eczema check… My eczema was very severe. My whole body was itchy, I scratched hard, and it bled everywhere. Recovery was very slow.” Such experiences often lead users to feel disappointed, anxious, or even averse. Overall, the negative attitudes of the Japanese public are mainly concentrated in three areas: skepticism about scientific validity, concerns about ecological ethics, and adverse personal experiences. These patterns reflect multiple cognitive and emotional challenges in the cross-cultural dissemination of TCM.

### Public behavioral interactions and intentions

#### Analysis of behavioral interactions

The previous section analyzed the focus of public attention, emotional trends, and the underlying causes. This section further examines how cognitive and affective factors influence public behavioral interactions and intentions.

To ensure the representativeness of the sample, we selected tweets with at least 10 replies, resulting in 697 posts for the behavioral interaction analysis. User engagement was measured across three dimensions (likes, replies, and retweets) through descriptive statistics. The analysis revealed that likes averaged 61.9(median = 31; CV = 335.2%), replies averaged 30.6 (median = 15; CV = 265.2%), and retweets averaged 32.7 (median = 17; CV = 266.4%). In all cases, the median was substantially lower than the mean, indicating that a small number of highly popular tweets disproportionately elevated the averages, reflecting the “head effect” a common phenomenon in social media dissemination. Furthermore, the high coefficients of variation across all engagement metrics indicate substantial volatility, suggesting that user attention is unevenly distributed across TCM-related topics and characterized by selective engagement. This pattern points to strong context dependence in the diffusion of TCM-related information among the Japanese public: rather than spreading uniformly, information propagates through localized, hotspot-driven dynamics.

**Figure 8:**
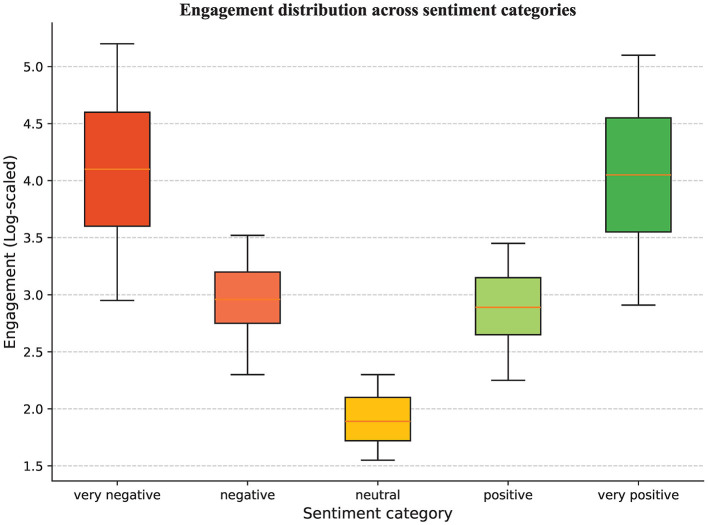
User engagement distribution across TCM-related sentiment categories. The engagement intensity of each tweet is defined as: *Eng*(*t*) = α•*Like*_*t*_+β•*Retweet*_*t*_+γ•*Reply*_*t*_, where *Like*_*t*_,*Retweet*_*t*_ and *Reply*_*t*_ denote the number of likes, retweets, and replies for tweet *t*, respectively. In this study, we assign weights of α = 0.25, β = 0.35, γ = 0.40. Aggregating the engagement intensities of tweets within each sentiment category yields the corresponding distribution of engagement intensity for that category.

As shown in Figure 8, tweet sentiment and user engagement exhibit a pronounced U-shaped relationship, with engagement increasing alongside sentiment intensity. Tweets expressing very positive or very negative sentiment attract significantly higher interaction than those with neutral or weak sentiment, with neutral content receiving the lowest engagement. This suggests that informational posts lacking affective cues are less effective at eliciting user participation. By contrast, emotionally charged content—irrespective of polarity—consistently generates higher levels of retweeting, replying, and other forms of interaction. These findings underscore the central role of sentiment intensity as a key driver of user engagement and social media diffusion.

A closer comparison across sentiment polarities reveals that engagement associated with very negative sentiment slightly exceeds that of very positive sentiment; more broadly, negative sentiment elicits higher levels of interaction than positive sentiment. This pattern suggests that, within TCM-related discourse, content highlighting “risk,” “side effects,” or “efficacy controversies” is more likely to capture attention and diffuse widely, indicating that negative sentiment carries greater diffusion potential. This finding aligns with prior research on the “negativity bias,” which posits that individuals are more sensitive to negative information and thus more likely to engage with and share it (44).

Taken together, the results show that sentiment intensity plays a central role in shaping user engagement, with stronger sentiment associated with higher levels of participation. Beyond serving as a byproduct of cognitive content, sentiment operates as a key mediating mechanism within the topic–engagement nexus: distinct topics elicit differentiated emotional responses, which in turn shape both the magnitude and form of user engagement. These findings provide strong empirical support for H2.

**Figure 9:**
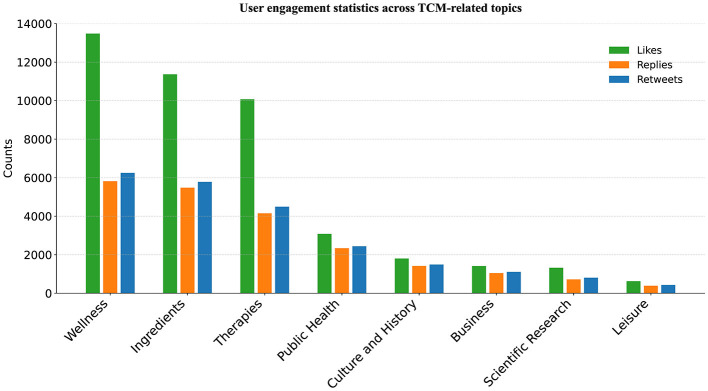
User engagement statistics across TCM-related topics.

Figure 9 presents the distribution of interaction metrics across topics. Building on these data, we conduct a quantitative analysis of user engagement behaviors (likes, replies, and retweets), revealing the Japanese public's cognitive preferences and interaction motivations regarding TCM-related topics. In general, the number of likes exceeded that of replies and retweets, indicating that TCM-related tweets attract considerable attention from the Japanese public, and that most users prefer low-cost interactions such as liking after reading the posts. The “Wellness” topic, in particular, received the highest number of likes. Notably, lifestyle-oriented posts, including those on the popularity of “herbal coffee” and “herbal mooncake” in China, received 4,010 and 2,726 likes, respectively, underscoring the strong public interest in, and cultural affinity for, the incorporation of TCM into everyday dietary practices. By contrast, topics focused on herbal components, therapeutic practices elicit relatively higher levels of replying and retweeting. This pattern indicates a strong desire among Japanese users to engage in discussions regarding the scientific basis and functional properties of TCM ingredients.

Across topic clusters, “Wellness,” “Ingredients,” and “Therapies” consistently attract the highest levels of engagement, with interaction intensity far exceeding that of other categories. This finding suggests that users exhibit strong identification with—and a high propensity to share—content related to acupuncture, Kampo ingredients, and everyday health practices, linking personal health needs to social media engagement. By contrast, participation in more specialized topics such as “Scientific Research” and “Public Health” remains comparatively low, with all engagement metrics trailing those of lifestyle-oriented topics. This divergence indicates that, although these domains are central to the modernization of TCM and public health governance, their technical complexity and higher cognitive demands constrain their ability to generate broad engagement. More broadly, the pattern reflects a tendency for users to ground both information selection and engagement behavior in everyday experience.

From the perspective of engagement structure, topics display distinct patterns across likes, replies, and retweets, with “Business” and “Public Health” characterized by notably higher reply-to-like ratios. This pattern suggests that, despite attracting smaller audiences and lower overall engagement, these topics are more likely to involve controversy or stakeholder salience, thereby eliciting more substantive forms of interaction beyond superficial “likes.”

Taken together, Japanese users' engagement with TCM-related discourse exhibits a stratified structure: lifestyle-oriented and highly pragmatic topics tend to generate high-volume but low-intensity interactions (e.g., likes), whereas more specialized or controversial topics elicit deeper forms of engagement, including discussion-oriented replies and diffusion-oriented retweets. These findings highlight substantial variation in public attention across topics and reveal differentiated patterns of social media behavior. Collectively, they provide strong empirical support for H3, which posits that TCM-related topics vary significantly in both overall engagement intensity and interaction structure.

#### Analysis of active learning behavior intentions

**Figure 10:**
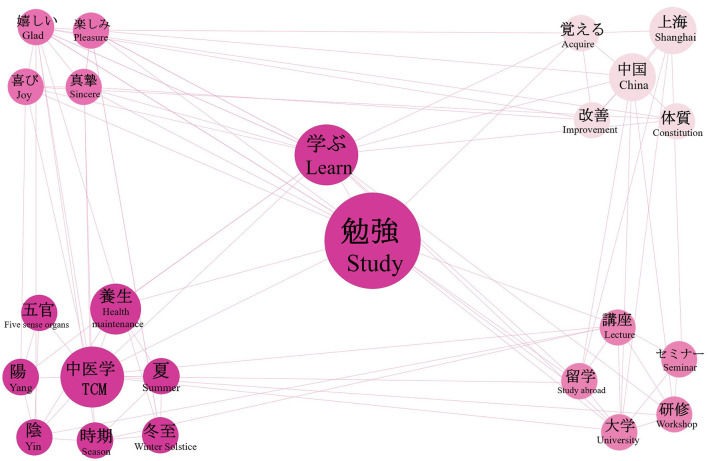
Semantic co-occurrence network graph of tweets on active learning behavior intention. In the figure, the size of each keyword node represents the frequency of the word and its average engagement intensity. When the *PMI*(*w, v*) coefficient between two words is greater than 0, the edge between word *w* and word *v* is retained.

Using “learn” and “study” as search terms indicative of active learning intentions, we identified relevant tweets and constructed a co-occurrence network (Figure 10). The associated vocabulary can be categorized into four dimensions: learning content, learning methods, learning motivations and goals, and emotional expressions. In terms of learning content, health maintenance and TCM are core elements of cross-cultural exchange and are highly valued by the Japanese public. Elements related to seasonal health practices, such as “five organs”, “winter solstice”, “Yin”, “Yang”, and “season”, also attract strong interest. In terms of learning methods, users often engage in systematic study through formal channels such as “university”, “seminar”, “workshop”, and “lecture”. Their learning goals extend beyond short-term motivations such as memorizing relevant TCM knowledge, encompassing longer term aspirations such as studying abroad (“China”, “Shanghai”) and improving personal health (“improvement”, “constitution”). Emotional expressions toward the learning subjects are primarily characterized by feelings of enjoyment (“glad”, “pleasure”, “joy”) and sincerity (“sincere”). Overall, Japanese public discourse on learning about TCM exhibits systematic and various features of active learning. Their learning content reflects strong interest in TCM theories and seasonal health culture; learning methods rely largely on formal and academic pathways; learning motivations combine short-term knowledge acquisition with long-term developmental goals; and emotional expressions reveal positive engagement and sincere exploration.

#### Analysis of cultural identity behavior intention

**Figure 11:**
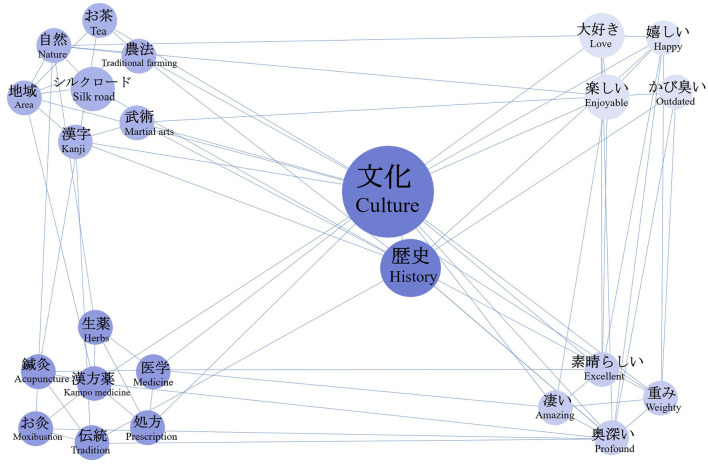
Semantic co-occurrence network graph of tweets on cultural identity behaveor intention.

Using “culture” and “history” as search terms to identify cultural identity intentions, we conducted a co-occurrence network analysis, the results of which are shown in Figure 11. The emergent clusters encompass traditional cultural elements, emotional resonance, and value evaluation. In addition to TCM-related cultural elements (“Kampo medicine”, “acupuncture”, “herbs”, “prescription”, “moxibustion”, “tradition”, “medicine”), the tweets also contain a wide range of Chinese cultural imagery, including natural aesthetics (“nature”, “traditional farming”, “area”), cultural exchange and heritage (“Silk Road”), and traditional cultural practices (“Chinese characters”, “martial arts”, “tea”). These elements of traditional Chinese culture trigger strong interest and emotional resonance among the Japanese public (“love”, “enjoyable”, “happy”), which further leads to highly positive evaluations of Chinese culture (“excellent”, “amazing”, “profound”, “weighty”).

However, these positive emotions do not represent a singular or uniform form of cultural identification. A notable counterpoint is that some users describe Chinese history as “old-fashioned” (かび臭い), revealing a dual psychological orientation within Japan's cross-cultural recognition process. On the one hand, they express respect and curiosity toward China's long history and rich cultural traditions; on the other hand, the negative perception suggests a continuation of impressions of “outdated” or “backward”. This ambivalence suggests that the historical weight of TCM and its associated cultural elements operates simultaneously as an attraction and a potential impediment in cross-cultural communication. Such contradictory attitudes underscore the dynamic character of cultural identification in the context of Sino–Japanese cultural exchange.

#### Analysis of experiential exploration behavior intention

**Figure 12:**
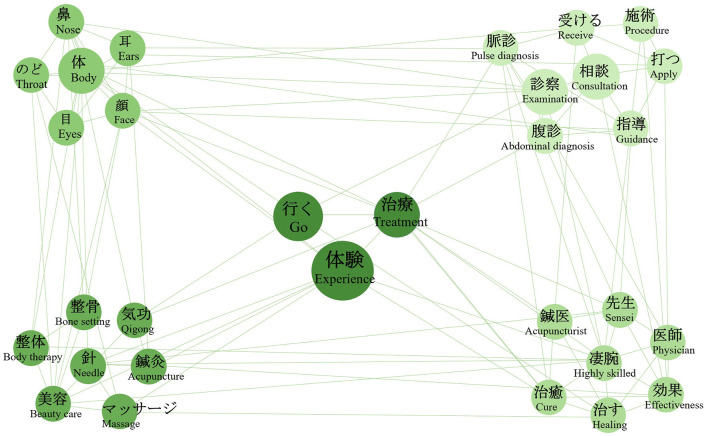
Semantic co-occurrence network graph of tweets on experiential exploration behavior intention.

Using “Experience”, “Treatment”, and “Go” as search terms to identify experiential exploration intentions, we performed a co-occurrence network analysis of Japanese tweets. As illustrated in Figure 12, the findings unfold across three interrelated dimensions: experience items, experiential processes, and subjective perceptions.

Regarding experience items, Japanese users show strong interest in options related to beauty and wellness, including “needle”, “massage”, “beauty care”, “seitai”, and “seikotsu.” These practices span traditional Chinese medical therapies while revealing an emerging convergence with contemporary beauty and wellness trends. Regarding the experiential process, users pay attention to the entire procedure from initial communication to hands-on treatment. This includes consultation and professional guidance (“consultation”, “instruction”); specific diagnostic methods in Chinese medicine (“pulse diagnosis”, “abdominal diagnosis”, “examination”); targeted body areas (“body”, “face”, “nose”, “throat”, “eyes”, “ears”); and specific treatment actions (“apply”, “receive”). This indicates the public's emphasis on professionalism and operational details throughout the experience. In terms of subjective perceptions, users evaluate both practitioner competence (“sensei”, “physician”, “acupuncturist”, “highly skilled”) and treatment effects and personal feelings (“effectiveness”, “cure”, “healing”). Therefore, the Japanese public's exploration of Chinese medical experiences demonstrates concerns across option selection, procedural operations, and expected perceptions.

## Discussion

Since the beginning of the 21st century, TCM as a form of “local knowledge” has gradually become an influential component of the global health system. Using large-scale data from Japanese social media, this study integrates topic modeling, sentiment analysis, and co-occurrence network analysis to investigate public concerns, sentiment orientations, and behavioral engagement patterns related to TCM, thereby elucidating the underlying mechanisms linking these three dimensions.

The empirical results reveal significant heterogeneity in sentiment expression across topics, with certain topics more likely to elicit positive or negative sentiment, supporting H1. Affective orientation significantly shapes user engagement and partially mediates the relationship between topics and interaction outcomes, supporting H2. Furthermore, topics differ substantially in both interaction intensity and structure, reflecting differentiated participation patterns and collectively supporting H3.

From the focus of public attention, the Japanese public is most concerned about clinical efficacy and daily wellness. Users tend to transform TCM knowledge into a tangible health-oriented lifestyle through personal experiences and narratives shared on social media. This “everyday narrative” not only enhances the cultural affinity of TCM but also exposes its limitations, particularly the uncertainty of its efficacy under Western scientific standards (4). On one hand, a substantial portion of users recognize TCM's benefits in disease prevention, chronic disease treatment, and health maintenance, supporting the TCM principle of “treating diseases before they arise.” On the other hand, a notable contingent questions TCM's scientific grounding and voices concerns over animal-derived ingredients and broader sustainability challenges.

The second noteworthy dimension is the relationship between traditional TCM knowledge and modern scientific research. Within the discursive frameworks of pandemic control and evidence-based medicine, the public reinterprets the modern significance of TCM, promoting its transformation from an experience-based medical tradition toward a science-oriented, evidence-seeking paradigm. Frequent mentions of terms such as “clinical trials” and “cultivation” signal that scientific rigor has become a key reference point through which users assess TCM's legitimacy. This shift shows that TCM faces challenges beyond efficacy debates, particularly how it can align traditional knowledge with scientific standards while preserving its cultural identity and global influence.

The third dimension of public attention is business and cultural promotion of TCM. Discussions about TCM enterprises and cultural products show that the image of TCM has increasingly been packaged as a cultural brand and media symbol, achieving “visualized” and “entertained” dissemination in consumer contexts. Through films, advertisements, and cultural products, TCM's historical imagery and Eastern aesthetics are presented, allowing the Japanese public to connect with Chinese culture.

Regarding public sentiment, the Japanese public generally holds a neutral to positive attitude toward TCM. The results of this study are consistent with Gao et al. (29), indicating that expressions of positive sentiment outweigh negative sentiment. However, compared with existing studies, this research further reveals that the sources of positive sentiments are more diverse. Specifically, Japanese users' positive attitudes arise not only from the enjoyment and functional expectations associated with daily TCM practices but also from TCM's cultural and entertainment significance. The popularity of Chinese films and related cultural products in Japan allows the public to encounter and recognize the symbolic significance of TCM during viewing and cultural consumption, generating emotional resonance.

Notably, while positive sentiment remains dominant, negative sentiment has steadily increased in the post-pandemic period. Although the overall proportion of negative sentiment is low, its content is complex and cannot be ignored. The Japanese public's negative sentiment mainly focuses on three factors: scientific skepticism, ecological and ethical issues, and adverse personal experiences. Firstly, TCM's reliance on empirical treatment methods and distinctive theoretical framework often invites skepticism regarding its efficacy and diagnostic consistency, with some users adopting a cautious or doubtful stance due to limited empirical validation. Meanwhile, ecological and ethical issues related to Chinese medicinal materials, such as overharvesting or animal protection concerns, also trigger public moral anxiety. Moreover, personal discomfort or lack of noticeable efficacy during TCM experiences can lead to disappointment or rejection. Such negative sentiment highlights trust and comprehension barriers in cross-cultural communication. It also underscores the need for future dissemination strategies that balance scientific validation, ecological and ethical transparency, and authentic experiential feedback. Compared with Western social media users (45), the Japanese public pays greater attention to both TCM and experiential dimensions, highlighting the uniqueness of emotional engagement and the role of media-induced cultural experiences in cross-national dissemination.

Regarding public behavioral engagement, Japanese users exhibit a progressive pattern of engagement that extends from online awareness to offline participation. Users often engage through low-cost actions such as likes and tend to participate in more in-depth interactions with tweets on TCM wellness, ingredients, and therapies. Content that combines everyday life and emotional aspects more easily elicits understanding, appreciation, and resonance from Japanese users. Beyond surface-level online interactions, social media content actively motivates tangible offline behaviors. Japanese users develop intentions to systematically study TCM knowledge, participate in cultural courses and lectures, actively explore Chinese traditional culture, and personally experience diverse practices such as acupuncture, tuina, qigong, and beauty cares. This demonstrates a deepening process from online interactive experience to offline participation.

Notably, the inclusion of keywords such as “Kampo” (漢方) broadens the scope of the retrieved corpus, capturing a wider spectrum of public discourse on traditional medicine. However, Kampo medicine and Traditional Chinese Medicine (TCM) share substantial commonalities in their underlying medical philosophy and clinical practice. From the perspective of public perception, the two systems are often regarded as closely related or overlapping traditions, and discussions involving “TCM” and “Kampo” exhibit a high degree of consistency in both sentiment expression and engagement behavior. While minor divergences may arise in specific instances, these remain limited and are unlikely to introduce systematic bias into the study's findings.

## Implications and limitations

### Theoretical implications

On one hand, this study advances the Cognition–Affect–Behavior (CAB) framework by extending its application to cross-cultural health communication and TCM-related discourse. By integrating cognition, affect, and behavioral engagement within a unified analytical framework, it elucidates their dynamic interdependencies in real-world communication contexts and enhances the model's explanatory scope and contextual applicability in digital media environments and non-Western medical systems.

On the other hand, the analytical framework proposed in this study reveals the structural connections among social media users' cognitive focus, emotional responses, and interactive behaviors in TCM-related discussions. It provides a clear path to understanding the analytical value of UGC in cultural cognition and cross-cultural communication research. The framework can be applied to cross-platform studies of tourism, film, translation, and other cultural products, providing a replicable tool for exploring the mechanisms of digital public cultural engagement.

### Policy implications

Based on the research findings, this study proposes strategic recommendations for enhancing the cross-cultural dissemination of TCM. The recommendations are as follows:

First, in response to public concerns regarding the scientific validity and safety of TCM, dissemination strategies should be coordinated at governmental, academic, and corporate levels. Governments can establish multi-stakeholder coordination mechanisms, integrating regulatory, media, and academic resources to enhance their capacity to respond to and guide international public opinion. Academic institutions should focus on the strengths of TCM, enhance collaboration with leading international medical institutions, and promote the publication of high-quality research in global outlets, thereby enhancing the authority and international visibility of TCM's scientific foundation. Corporations should enforce stringent standards for medicinal material sourcing and quality control, while incorporating TCM cultural symbols into media campaigns and cultural consumer experiences to boost brand visibility, strengthen public trust, and improve the effectiveness of international dissemination.

Second, in response to the finding that the public emphasizes cultural and experiential dimensions, efforts should be made to actively develop the digital TCM cultural consumption sector. This includes enhancing accessibility and the immersive experience of cultural offerings. Specific measures include developing virtual experiential courses, online lectures, and interactive health management apps that closely integrate TCM theory with the public's daily life and health needs, allowing users to gain knowledge, practical skills, and hands-on experience through active participation. At the same time, through media representations such as films, cultural products, advertisements, and exhibitions, the historical imagery of TCM can be transformed into culturally accessible and recognizable content. This approach enhances emotional resonance and cultural identity, thereby fostering the sustained influence of TCM in digital contexts.

Finally, to foster public behavioral intentions, strategies integrating online and offline activities can further promote tangible experiences and interactions. Online engagement can be stimulated through social media short videos, live streaming, health science platforms, and interactive Q&A. Additionally, online communities and challenges help maintain sustained interaction and peer-to-peer dissemination. Offline, initiatives such as study camps, experiential courses, cultural exhibitions, and TCM-themed tourism activities enable audiences to actively engage in diverse TCM cultural experiences, facilitating the transition from awareness to active participation.

### Limitations and future directions

Although the results of this study effectively address the three research questions, there are certain limitations in the data and methodology. First, a key limitation of this study lies in the relatively broad scope of keyword selection used for data collection. Although this approach enhances corpus coverage, it may also introduce discourse associated with Japan's indigenous traditional medical system through the inclusion of terms such as “Kampo” and “traditional medicine.” Future research could address this issue by implementing more fine-grained keyword design to better delineate the conceptual boundaries of the corpus. Furthermore, multilingual comparative analyzes would help assess the robustness of the findings across cultural contexts and facilitate the identification of latent micro-level variations. Second, data representativeness is limited. This study relies solely on Twitter data and does not include other major international social media platforms such as YouTube, Reddit, Facebook, and TikTok. Short-video platforms, particularly TikTok, have become increasingly important venues for public expression, hosting large volumes of online discourse. The absence of such data may, to some extent, overlook the characteristics of video-oriented users and potentially limit the generalizability of the findings. Future research should expand both the dataset and methodological framework by incorporating short-video platform data into a unified analytical architecture. Furthermore, cross-platform comparative studies could examine how platform-specific affordances shape cultural diffusion processes and the formation of public attitudes. Finally, user information is limited. Although posting content and interaction behaviors were collected, personal information such as age, gender, education level, and income could not be obtained. These factors may considerably influence public perceptions and attitudes toward TCM. Future research could incorporate richer user-level data to examine heterogeneity across population groups in the cognitive, affective, and behavioral dimensions of engagement with TCM-related discourse. This would enable a more comprehensive understanding of public cognition, sentiment, and behavioral patterns, and provide more precise evidence for both academic inquiry and policy-making.

## Conclusions

Grounded in the Cognition–Affect–Behavior (CAB) theoretical framework, this study employs advanced text mining techniques—including topic modeling, sentiment analysis, and co-occurrence network analysis— to systematically examine public attitudes toward TCM in Japan. In doing so, it provides a comprehensive account of the mechanisms underlying public discourse on TCM.

At the cognitive level, topic modeling shows that public attention is primarily concentrated on “Therapies,” “Wellness,” and “Ingredients,” reflecting underlying knowledge structures and informational preferences regarding TCM. At the affective level, sentiment varies systematically across topics: efficacy- and wellness-related discussions are predominantly positive or neutral, whereas content concerning safety and ethical controversies tends to elicit negative sentiment, highlighting the influence of cognitive content on emotional responses. At the behavioral level, engagement behaviors—including likes, replies, and retweets—display pronounced topic dependency, indicating that informational attributes shape both the intensity and form of participation and diffusion. Taken together, these findings validate the CAB framework by demonstrating a sequential pathway in which cognition shapes affect and affect drives behavior. They further show that public engagement with TCM in Japan is structurally grounded rather than stochastic, providing robust theoretical and empirical insights into the dynamics of health-related discourse diffusion on social media.

At the theoretical level, this study elucidates the cognition, sentiment, and behavior of the Japanese public in engaging with TCM, addressing a critical gap in research on public perceptions. The results reveal that the cross-cultural transmission of TCM extends beyond medical cognition and scientific validation, which are profoundly influenced by cultural symbolism, media experience, and interactive behavior. These findings enrich theoretical discussions on cultural transmission and cross-cultural identity construction, providing novel perspectives and analytical frameworks for future research.

At the practical level, this study provides empirical evidence to support governments, institutions, and enterprises in formulating effective international communication strategies for TCM. By analyzing Japanese public cognition, sentiment, and behavior in detail, it offers valuable insights for targeted communication and strategic market positioning of TCM-related industries. Moreover, the proposed data-driven analytical framework demonstrates the feasibility of extracting fine-grained insights from large-scale social media data, offering methodological implications for future research on digital health communication.

## Data Availability

The datasets presented in this study can be found in online repositories. The names of the repository/repositories and accession number(s) can be found in the article/Supplementary material.

## References

[1] Xinhua News Agency. Xi Jinping stresses TCM as a “treasure of Chinese civilization.” (2024). Available online at: https://www.gov.cn/yaowen/liebiao/202412/content˙6990838.htm?utm˙source=chatgpt.com (Accessed February 10, 2026).

[2] Croizier RC. Traditional medicine in modern China: Science, nationalism, and the tensions of cultural change. Harvard University Press. (1968). p. 81–104. doi: 10.4159/harvard.9780674430686

[3] Eigenschink M Dearing L Dablander TE Maier J Sitte HH. A critical examination of the main premises of Traditional Chinese Medicine. Wien Klin Wochenschr.(2020) 132:260–73. doi: 10.1007/s00508-020-01625-w 32198544 PMC7253514

[4] Wang N Xu J. Speaking modernity: From the “Debate between Traditional Chinese Medicine and Western medicine” to the “Debate between science and metaphysics”: A study of media discourse based on the “proposal of abolishing Traditional Chinese Medicine” in the Republic of China. J Med Humanity Media. (2024) 2:79–100. doi: 10.62787/mhm.v2i3.58

[5] Liu J Zhang L. Affective polarization in online cross-cutting discussions about traditional Chinese Medicine: national identity's moderation effect. Chin J Commun.(2025) 1–21. doi: 10.1080/17544750.2025.2504367

[6] McCartney G Wang CF Peng Y. Betting on traditional Chinese medicine (TCM) within medical tourism (MT) development: the case of Macao. Tour Recreat Res.(2024) 50:1–7. doi: 10.1080/02508281.2024.2424985

[7] Liu S Zhao Y. “Exploration of the Application of Virtual Exhibition Technology in the Dissemination of Traditional Chinese Medicine Culture.,” In:Stephanidis C Antona M Ntoa S Salvendy G, editors. HCI International 2025 Posters. Communications in Computer and Information Science. Cham: Springer Nature Switzerland (2025). p. 58–68 doi: 10.1007/978-3-031-94165-8_7

[8] Kayo T Uneda K Suzuki M. Acupuncture topics on twitter (Currently X) in English and Japanese: Co-occurrence Network Analysis. Cureus.(2024) 16:e54928 doi: 10.7759/cureus.54928 PMC10966937 38544657

[9] Okawa Y Ideguchi N Yamashita H. Relationship between health literacy and attitudes toward acupuncture: a web-based cross-sectional survey with a panel of Japanese residents. PLoS ONE. (2023) 18:e0292729. doi: 10.1371/journal.pone.0292729 37862311 PMC10588898

[10] Lyu S Zhao Z Liu G Zhou S. Understanding Embodied Experiences in a Traditional Chinese Medicine-Based Health Promotion Program: Insights from In-Depth Interviews and Participant Observations. Health Commun.(2025) 41:1–11. doi: 10.1080/10410236.2025.2490230 40256996

[11] Peng AY Chen S. Traditional Chinese medicine works: a politicised scientific debate in the COVID-19 pandemic. Asian J Commun.(2021) 31:421–35. doi: 10.1080/01292986.2021.1913618

[12] Shen J Xu D. Cyberbalkanization without monotonic polarization: temporal dynamics and user heterogeneity in online debates on traditional Chinese medicine. Soc Sci Comput Rev.(2024) 43:1306–26. doi: 10.1177/08944393241301043

[13] Wang D Lu J Zhou J Wong VKW. Useful or not? The discussion of traditional Chinese medicine to treat COVID-19 on a Chinese social networking site. BMJ Glob Health. (2024) 9:e014398. doi: 10.1136/bmjgh-2023-014398 PMC11168149 38857946

[14] Goldsmith BE Horiuchi Y Matush K. Does public diplomacy sway foreign public opinion? Identifying the effect of high-level visits. Am Polit Sci Rev.(2021) 115:1342–57. doi: 10.1017/S0003055421000393

[15] Lyu S Zhao Z Liu G Zhou S. Understanding embodied experiences in a traditional Chinese medicine-based health promotion program: insights from in-depth interviews and participant observations. Health Commun.(2025) 41:52–62. doi: 10.1080/10410236.2025.2490230 40256996

[16] Zhao H Zhang R Chen Y. The influencing role of cultural values on attitudes of the chinese public towards traditional Chinese Medicine (TCM) for the Control of COVID-19. Patient prefer adherence. Volume.(2023) 17:3589–605. doi: 10.2147/PPA.S443713 PMC10759415 38169962

[17] Wu Z He Q Li J Bi G Antwi-Afari MF. Public attitudes and sentiments towards new energy vehicles in China: a text mining approach. Renew Sustain Energy Rev.(2023) 178:113242. doi: 10.1016/j.rser.2023.113242

[18] Chen L Wu X Li M. Formation and fragmentation within a networked public sphere: social media debates on Traditional Chinese Medicine. Telemat Inform. (2018) 35:2219–31. doi: 10.1016/j.tele.2018.08.008

[19] Zhang W He Y Min Y. Intermedia emotional agenda-setting and cultural distance: a cross-cultural analysis of global coverage of traditional Chinese medicine (2018–2024). Int Commun Chin Cult.(2025) 13:1–29. doi: 10.1007/s40636-025-00342-1

[20] GROSSBERG L. Mediamaking: Mass media in a popular culture. 2nd ed. Thousand Oaks, CA: Sage Publications, Inc (2006).

[21] Shim J-M. The influence of social context on the treatment outcomes of complementary and alternative medicine: the case of acupuncture and herbal medicine in Japan and the U.S. Glob Health.(2015) 11:17. doi: 10.1186/s12992-015-0103-2 PMC4415294 25907272

[22] Kusudo M Terada M Kureyama N Wanifuchi-Endo Y Fujita T Asano T . Characterizing user demographics in posts related to breast, lung and colon cancer on Japanese twitter (X). Sci Rep.(2024) 14:6485. doi: 10.1038/s41598-024-56679-x 38499598 PMC10948868

[23] Suzuki T Tanimoto T Kamamoto S Ozaki A Torii HA Hase D . Characteristics of Japanese physician influencers on Twitter during the COVID-19 pandemic and fact-checking their tweets on COVID-19-related drugs. Postgrad Med J.(2024) 100:91–5. doi: 10.1093/postmj/qgad098 37968828

[24] Shifman L. Cross-cultural comparisons of user-generated content: an analytical framework. Int J Commun.(2016) 10:5644–63.

[25] Lavidge RJ Steiner GA. A model for predictive measurements of advertising effectiveness. J Mark.(1961) 25:59–62. doi: 10.1177/002224296102500611

[26] Zhu G. A neoliberal transformation or the revival of ancient healing? A critical analysis of traditional Chinese medicine discourse on Chinese television. Crit Public Health. (2022) 32:689–99. doi: 10.1080/09581596.2021.1919290

[27] Zhou F. Traditional Knowledge, science and China's pride: how a TCM social media account legitimizes TCM treatment of COVID-19. Soc Semiot.(2023) 33:697–713. doi: 10.1080/10350330.2021.1926964

[28] Duan G Li J Li S Dai D Yu H. Investigation and countermeasure research on perceived value and willingness to medical treatment of traditional Chinese Medicine Service by the Public. DEStech Trans Econ Bus Manag.(2017) 641–6. doi: 10.12783/dtem/icem2017/13195

[29] Gao H Guo D Wu J Li L. Weibo users' emotion and sentiment orientation in traditional Chinese Medicine (TCM) during the COVID-19 pandemic. Disaster Med Public Health Prep.(2022) 16:1835–8. doi: 10.1017/dmp.2021.259 34369351 PMC8505818

[30] Bai H Yu G. A Weibo-based approach to disaster informatics: incidents monitor in post-disaster situation via Weibo text negative sentiment analysis. Nat Hazards.(2016) 83:1177–96. doi: 10.1007/s11069-016-2370-5

[31] World Population Review. Twitter users by country 2025. (2025). Available online at: https://worldpopulationreview.com/country-rankings/twitter-users-by-country?utm˙source=chatgpt.com (Accessed February 10, 2026).

[32] Grootendorst M. BERTopic: neural topic modeling with a class-based TF-IDF procedure. arXiv [preprint]. (2022). doi: 10.48550/arXiv.2203.05794

[33] Murayama T Miyazaki K Matsubara Y Sakurai Y. Linguistic landscape of generative ai perception: a global twitter analysis across 14 languages. (2025) 19:1262–94. doi: 10.1609/icwsm.v19i1.35872

[34] Veigel N Kreibich H De Bruijn JA Aerts JCJH Cominola A. Content analysis of multi-annual time series of flood-related Twitter (X) data. Nat Hazards Earth Syst Sci.(2025) 25:879–91. doi: 10.5194/nhess-25-879-2025

[35] Reimers N Gurevych I. Sentence-BERT: sentence embeddings using Siamese BERT-Networks. Proc 2019: Conference on Empirical Methods in Natural Language Processing and the 9th International Joint Conference on Natural Language Processing.(2019) 3982–92. doi: 10.18653/v1/D19-1410

[36] McInnes L Healy J Melville J. UMAP: uniform manifold approximation and projection for dimension reduction. arXiv [preprint]. (2020). doi: 10.48550/arXiv.1802.03426

[37] McInnes L Healy J Astels S. hdbscan: Hierarchical density based clustering. J Open Source Softw.(2017) 2:205. doi: 10.21105/joss.00205

[38] Liu J Li K Zhu A Hong B Zhao P Dai S . Application of Deep Learning-Based Natural Language Processing in Multilingual Sentiment Analysis. Mediterr J Basic Appl Sci. (2024) 8:243–60. doi: 10.46382/MJBAS.2024.8219

[39] Pletikosa Cvijikj I Michahelles F. Online engagement factors on Facebook brand pages. Soc Netw Anal Min.(2013) 3:843–61. doi: 10.1007/s13278-013-0098-8

[40] Bandura A. Social cognitive theory of mass communication. Media Psychol. (2001) 3:265–99. doi: 10.1207/S1532785XMEP0303_03

[41] Kim YY. Becoming intercultural: An integrative theory of communication and cross-cultural adaptation. Thousand Oaks, CA: Sage Publications, Inc (2001). doi: 10.4135/9781452233253

[42] Leydesdorff L Welbers K. The semantic mapping of words and co-words in contexts. J Informetr.(2011) 5:469–75. doi: 10.1016/j.joi.2011.01.008

[43] Goffman E. The Presentation of Self in Everyday Life. Scotland: Doubleday (1956). 251 p.

[44] Rozin P Royzman EB. Negativity bias, negativity dominance, and contagion. Personal Soc Psychol Rev.(2001) 5:296–320. doi: 10.1207/S15327957PSPR0504_2

[45] Lam CS Zhou K Loong HH-F Chung VC-H Ngan CK Cheung YT. The use of traditional, complementary, and integrative medicine in cancer: data-mining study of 1 million web-based posts from health forums and social media platforms. J Med Internet Res.(2023) 25:e45408. doi: 10.2196/45408 37083752 PMC10163397

