# Supplementary material for: Public perceptions and engagement with Traditional Chinese Medicine on Japanese social media (2010–2025): a text mining approach
Source: Front Public Health. 2026 May 19;14:1826864. doi: 10.3389/fpubh.2026.1826864 (PMC13226619; doi:10.3389/fpubh.2026.1826864)
Supplement: Supplementary file 1 [file Data_Sheet_1.docx]

**Distribution of TCM-related Tweets Across Topics**

| **Topic** | **Tweets Num** |
| --- | --- |
| Wellness | 8060 |
| Ingredients | 6282 |
| Therapies | 9647 |
| Public Health | 5356 |
| Culture and History | 3700 |
| Business | 2180 |
| Scientific Research | 2842 |
| Leisure | 1520 |

**User engagement statistics across TCM-related topics**

| **Topic** | **Likes** | **Replies** | **Retweets** |
| --- | --- | --- | --- |
| Wellness | 13482 | 5817 | 6251 |
| Ingredients | 11364 | 5481 | 5782 |
| Therapies | 10070 | 4149 | 4494 |
| Public Health | 3085 | 2341 | 2442 |
| Culture and History | 1806 | 1419 | 1490 |
| Business | 1412 | 1047 | 1106 |
| Scientific Research | 1320 | 715 | 805 |
| Leisure | 625 | 386 | 431 |
